# Supplementary material for: Atomic resolution dynamics of cohesive interactions in phase-separated Nup98 FG domains
Source: Nat Commun. 2022 Mar 21;13:1494. doi: 10.1038/s41467-022-28821-8 (PMC8938434; doi:10.1038/s41467-022-28821-8)
Supplement: Supplementary file 1 — Supplementary Information [file 41467_2022_28821_MOESM1_ESM.pdf]

Supplementary Information:

Atomic resolution dynamics of cohesive interactions in phase-separated Nup98 FG domains

Eszter Éva Najbauer[1], Sheung Chun Ng[2], Christian Griesinger[1], Dirk Görlich[2] & Loren B. Andreas[1]

[1]Department of NMR-based Structural Biology,
[2]Department of Cellular Logistics,
Max Planck Institute for Multidisciplinary Sciences, Göttingen, Germany

To whom correspondence should be addressed: E-mail: goerlich@mpinat.mpg.de (DG)/ land@mpinat.mpg.de (LBA)

Contents:

**Supplementary Figures 1-7** with legends	Page 2-9
**Supplementary Note 1:** Amino acid sequences of FG domain variants and a wild type Nup98 FG domain	Page 10-13
**Supplementary Tables 1-2**	Page 14-15
**References**	Page 16

Supplementary Figures

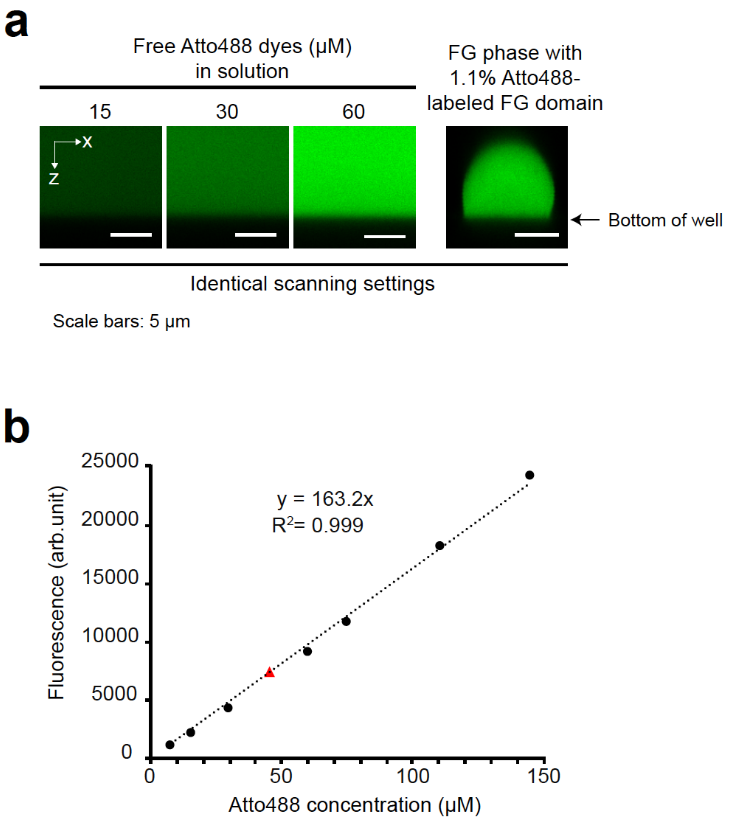

Supp. Fig. 1 - Measurement of intra-FG phase concentration.

(a) 2 µl of FG domain stock containing 900 µM uncoupled prf.GLFG$_{52x12}$ and 10 µM Atto488-coupled prf.GLFG$_{52x12}$ in 2 M guanidine hydrochloride was rapidly diluted with 100 µl assay buffer (20 mM NaPi pH 6.8, 150 mM NaCl, 5 mM DTT) and 30 µl of the mixture, containing the assembled FG phase, was placed on a micro-slide well for confocal microscopy. 1.1% of the FG domain molecules in the assembled FG phase contain an Atto488-fluorophore, assuming an even distribution of the molecules. In parallel, a concentration series of a standard, free Atto488-maleimide (which had been quenched with free L-cysteine as described[5]) in the same buffer was imaged. Representative confocal XZ-scans (showing the longitudinal dimension) of the FG phase and the standard series are shown. **(b)** Atto488-fluorescence signals of the standard series (black circles) and within the FG phase (red triangle) were quantified, and the Atto488 molar concentration within the FG phase can be determined from the standard curve (=46 µM). Therefore, total intra-phase FG domain concentration =46 µM/ 1.1% = 4180 µM ~4.2 mM (240 mg/ml protein).

This experiment was repeated three times. The mean total intra-phase FG domain concentration was 4.5 mM, with S.D.= 0.3 mM.

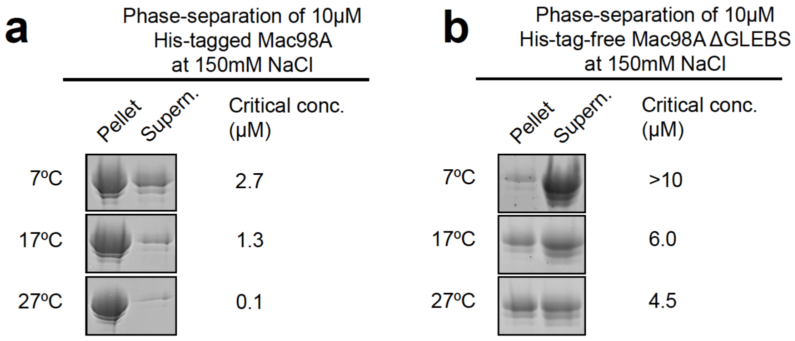

Supp. Fig. 2 - Temperature dependent phase separation of Mac98A FG domain.
(a) Mac98A FG domain was expressed as a polyhistidine-tagged version. Critical concentrations for phase separation of this construct decrease with increasing temperature (LCST behaviour). **(b)** We also constructed another version (His-tag-free Mac98A ΔGLEBS) which lacks the polyhistidine-tag and GLEBS domain. This construct is more comparable to prf.$GLFG_{52x12}$ in this study. This construct also shows LCST behaviour. Full scans of gels with molecular weight markers are provided in the Source Data file.

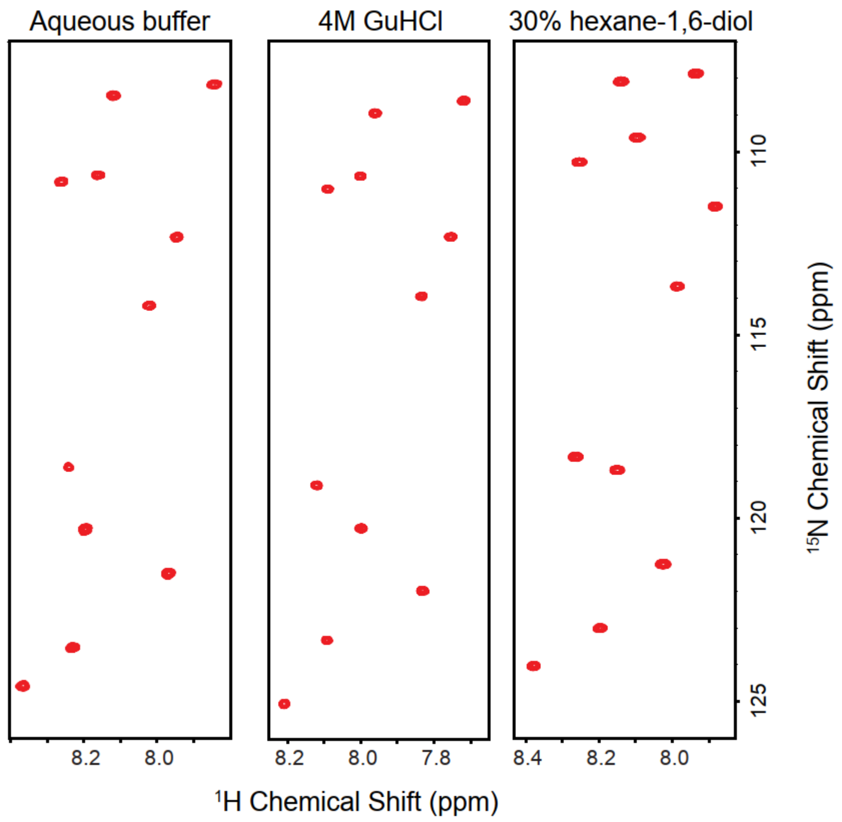

Supp. Fig. 3 - HSQC spectra of prf.GLFG$_{52x12}$ are minimally affected by denaturing conditions.

^{15}N-^{1}H HSQC spectra of the soluble state prf.GLFG$_{52x12}$ in an aqueous buffer (20 mM NaPi pH 6.8, 150 mM NaCl), as well as in two denaturing agents (which prohibit phase separation): 4 M guanidine hydrochloride (GuHCl) and 30% hexane-1,6-diol. The dispersion of ^{1}H chemical shifts is practically constant regardless of the environment, showing that the protein is disordered even under non-denaturing conditions (also in the FG phase, see Figure 2 in the main text).

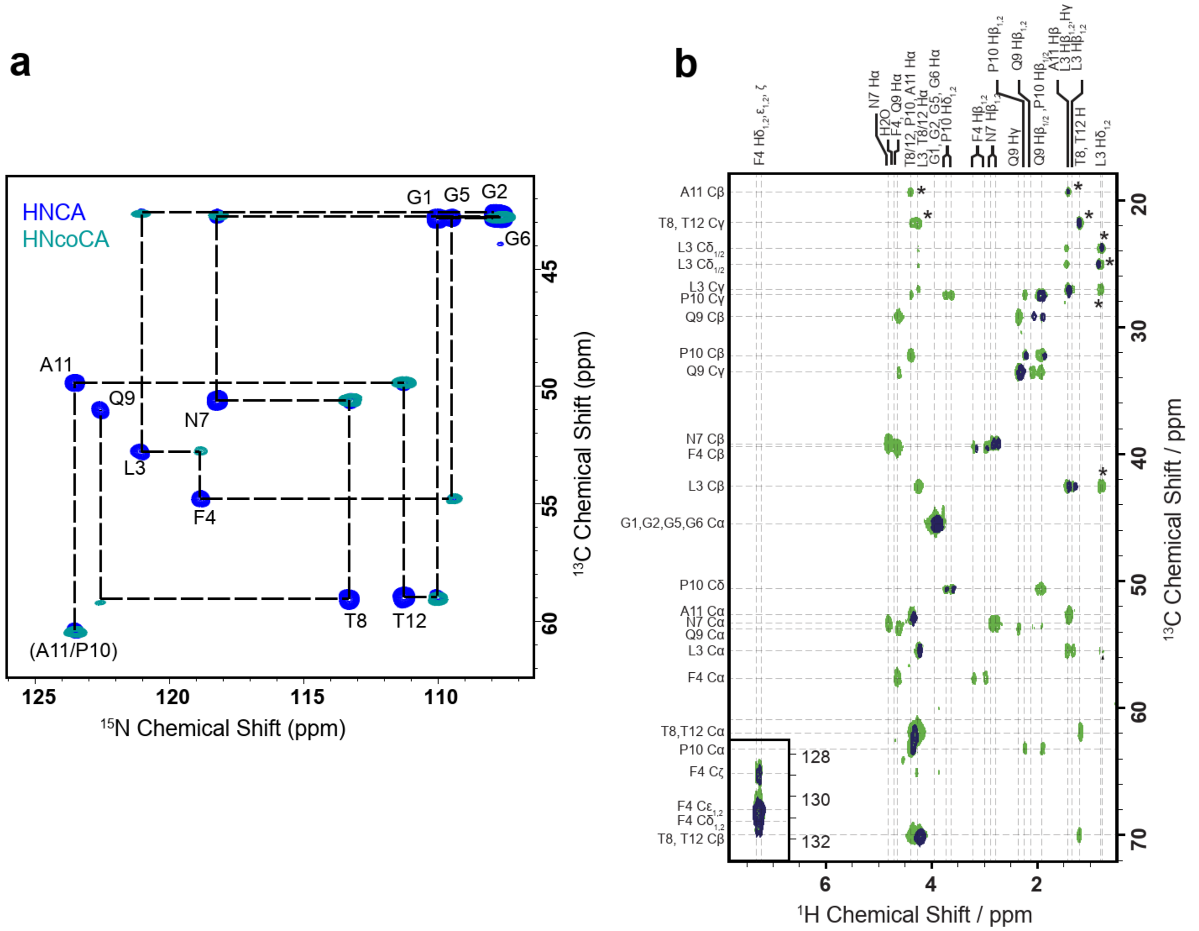

Supp. Fig. 4 - Assignment of the protein backbone and sidechains.

(a) Assignment of the backbone resonances of a repeat of prf.GLFG$_{52x12}$ in the FG phase. ^{13}C-^{15}N projection of the HNCA and the HN(CO)CA spectra. The backbone walk is shown with dashed lines. The backbone walk is broken due to the presence of proline in the sequence. Since the sequence is perfectly repetitive, a link between T12 and G1 can be observed. **(b)** Assignment of prf.GLFG$_{52x12}$ sidechains. Mixing of magnetization between the carbons of the same spin system was achieved using the WALTZ[1,2] scheme. The resulting (H)C(C)H spectrum is shown in light green, in dark blue, the ^{13}C-^{1}H HSQC^{SSMAS} one-bond correlation spectrum. Both spectra were recorded at 36 °C. Dashed lines are used to guide the eye to the chemical shift values of the assigned ^{13}C resonances (left) as well as ^{1}H resonances (top). In case of overlapping chemical shifts, all possibilities are listed. Peaks marked with an asterisk (*) are shown at 5x contour levels compared to the rest of the spectrum. The inset shows the aromatic F4 sidechain. The (H)C(C)H spectrum is contoured at 2x compared to the contours used for the aliphatic peaks. The strong water signal has been removed from the ^{13}C-^{1}H HSQC^{SSMAS} correlation spectrum to allow observation of the Hα resonances clearly visible in the (H)C(C)H spectrum, but its position has been marked among the ^{1}H chemical shift values.

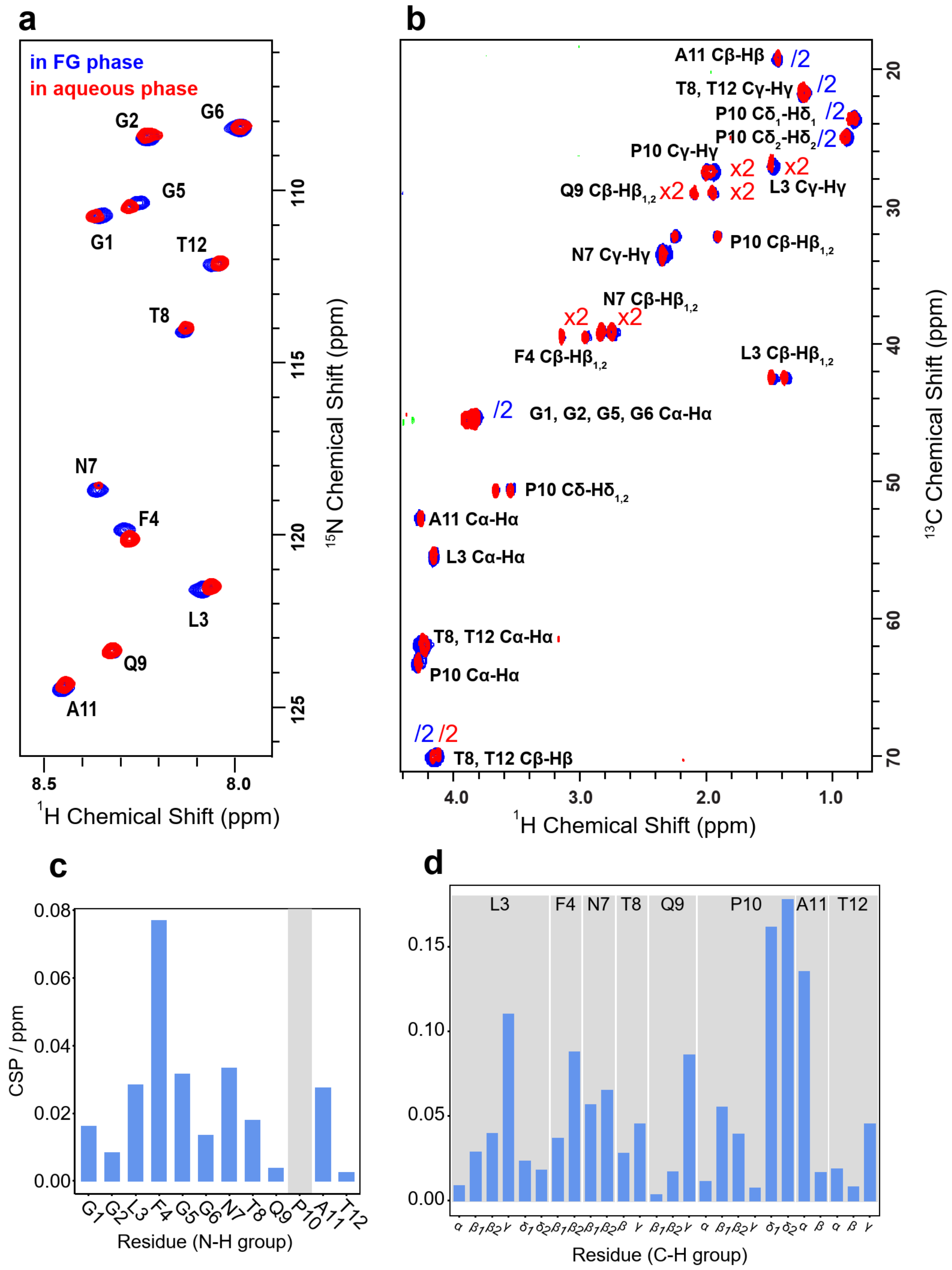

(**Supp. Fig. 5**, legend on the next page)

Supp. Fig. 5 - Chemical shift changes upon phase separation.

^{15}N-^{1}H **(a)** and ^{13}C-^{1}H **(b)** correlation spectra of both states (soluble state and FG phase) of prf.GLFG$_{52x12}$ measured at the same temperature (24 °C) and in the same buffer, and overlaid to observe spectral changes upon phase separation. Due to the large dynamic range in peak intensities, some weaker peaks are scaled up by a factor of two (i.e. shown at 2-fold lower contour levels (x2)), whereas some intense peaks are scaled down by a factor of two (/2). In (a) a small systematic shift in the ^{1}H dimension (stemming most probably from temperature miscalibration) was corrected by the average of chemical shift differences in the ^{1}H dimension. Chemical shift perturbations calculated for both ^{15}N-^{1}H **(c)** and ^{13}C-^{1}H **(d)** correlation spectra according to Williamson[3]. In (d), chemical shift perturbations were calculated only for groups that could be unambiguously assigned (do not suffer from signal overlap). Chemical shift perturbations were calculated separately for each peak in the spectrum (in case of diasteretopic protons or methyl groups these are given as separate values). Peaks arising from diastereotopic protons or methyl groups are denoted with 1 and 2.

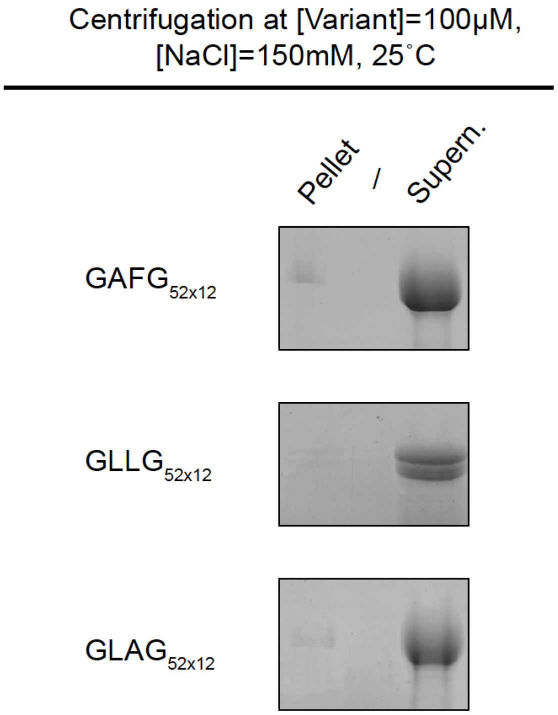

Supp. Fig. 6 - GAFG$_{52x12}$, GLLG$_{52x12}$ and GLAG$_{52x12}$ do not phase separate up to 100 µM. 100 µM dilutions of indicated FG domain variants were prepared and centrifuged at 25 °C at [NaCl]=150 mM. SDS samples of the contents pelleted (if any) and supernatants (soluble contents) were diluted further and loaded for SDS-PAGE at equivalent ratio (each 0.6% of the total), followed by Coomassie blue staining, and the gels are shown. Note that essentially no phase-separated material was detected for the variants tested herein. Full scans of gels with molecular weight markers are provided in the Source Data file.

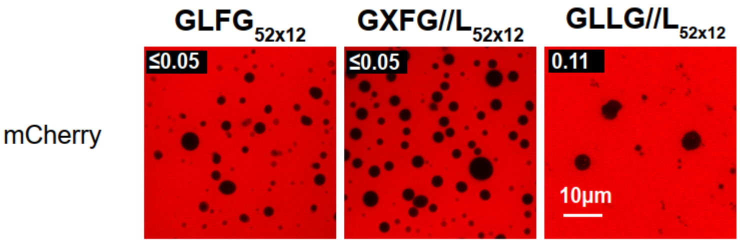

Supp. Fig. 7 - A phenylalanine-free but leucine-rich variant phase separates into near-spherical particles that exhibit barrier-like property.

Indicated FG domain variants were dissolved at 1 mM concentration in 4 M guanidine hydrochloride, and phase separation was initiated by a rapid 50-fold dilution with a buffer containing 50 mM Tris/HCl pH 7.5, 150 mM NaCl, 5 mM DTT, followed by another 4-fold dilution in buffer +6 µM mCherry. Samples were analysed by confocal laser scanning microscopy, resulting images are shown. All FG domain variants, including the Phe-free GLLG//L_{52x12}, phase separated to µm-sized, spherical particles that exclude mCherry protein (red) very well (the numbers indicate the partition coefficients of mCherry).

Supplementary Note 1:
Amino acid sequences of FG domain variants and a wild type Nup98 FG domain

Coloured in blue: polyhistidine-tag (30 residues)
Coloured in red: GLEBS (GLE2-binding-sequence) domain (44 residues)
For those marked with #: an alternative construct was used for Atto488-labelling/ FRAP experiments, see Supp. Table 1 for details.

Wild type *Tetrahymena thermophila* macronuclear Nup98A (wt Mac98A) FG domain
Plasmid: pHBS418

MQHHSHHGHHSHHGHHGHHGHHGHHGHHGSMFGNTGGGGLFGNTQTQQTGGGLFGQPQQTQFGQTGATGGGLFGGAT
NTFGGGGGGGLFGGNNNQQTNPTAGGGIFGQGTTGLGGAPAQTGGGLFGAPQNNQGGGLFGGGTTTGGGMFGNQANT
QTGGGGLFGGPSQPTTQPPAFSLNNPTTGGGGLFGQPANTMGGNNGGLFGGQTNSFGANNNMLGNNNRPQGAGIFGG
ATTTAPTGNTGMFGGIGANNGGGGLFGMNNTNTNPTGGFGATNPTAGGGGLFGGGATTTGGGGLFGGGNTQGGGLLG
TANTTAGGLLGGGFNMNNNTGGILGQTNNQFGLGSFGTNNNAAAAPFQPKASANGVLTKPNEKNLCYAISNGTDFCI
FELALTQRKLVKAGQLKPGAQQAGGMFGQPAQGGNGLFGGGGAATTTPFGGAQNGNLFGGQNTQAQGGGLFGAPVNN
AATGAGGGLFGAKPAATTTGGGLFGQMPAQTGGFLGNTATQPAGGGLFGGATTTQAPGGGGGGGLFGGNTTAATTGG
GLFGGNTQTGGATGGLFGGQQPNNQGGLFLNTGNANNANTGGGLFGGATTTPATGGGLFGGSTNTQPGLATGGGLFG
NNQGASQPAAQGGLFGGAAPQQNSLFGGATAGGQTGGLFGGATGATQQQGGGLFGQTASNPTQGGGLFGAANPGLGG
AAATSC

prf.GLFG$_{52x12}$
Plasmid: pSNG064#

GSGGLFGGNTQPATGGLFGGNTQPATGGLFGGNTQPATGGLFGGNTQPATGGLFGGNTQPATGGLFGGNTQPATGGL
FGGNTQPATGGLFGGNTQPATGGLFGGNTQPATGGLFGGNTQPATGGLFGGNTQPATGGLFGGNTQPATGGLFGGNT
QPATGGLFGGNTQPATGGLFGGNTQPATGGLFGGNTQPATGGLFGGNTQPATGGLFGGNTQPATGGLFGGNTQPATG
GLFGGNTQPATGGLFGGNTQPATGGLFGGNTQPATGGLFGGNTQPATGGLFGGNTQPATGGLFGGNTQPATGGLFGG
NTQPATGGLFGGNTQPATGGLFGGNTQPATGGLFGGNTQPATGGLFGGNTQPATGGLFGGNTQPATGGLFGGNTQPA
TGGLFGGNTQPATGGLFGGNTQPATGGLFGGNTQPATGGLFGGNTQPATGGLFGGNTQPATGGLFGGNTQPATGGLF
GGNTQPATGGLFGGNTQPATGGLFGGNTQPATGGLFGGNTQPATGGLFGGNTQPATGGLFGGNTQPATGGLFGGNTQ
PATGGLFGGNTQPATGGLFGGNTQPATGGLFGGNTQPATGGLFGGNTQPATGGLFGGNTQPATGGLFGGNTQPATGG
LFGGNTQPATS

Pro-free prf.GLFG$_{52x12}$
Plasmid: pSNG037

MQHHSHHGHHSHHGHHGHHGHHGHHGHHGSGGLFGGATNSQTGGLFGGATNSQTGGLFGGATNSQTGGLFGGATNSQ
TGGLFGGATNSQTGGLFGGATNSQTGGLFGGATNSQTGGLFGGATNSQTGGLFGGATNSQTGGLFGGATNSQTGGLF
GGATNSQTGGLFGGATNSQTGGLFGGATNSQTGGLFGGATNSQTGGLFGGATNSQTGGLFGGATNSQTGGLFGGATN
SQTGGLFGGATNSQTGGLFGGATNSQTGGLFGGATNSQTGGLFGGATNSQTGGLFGGATNSQTGGLFGGATNSQTGG
LFGGATNSQTGGLFGGATNSQTGGLFGGATNSQTGGLFGGATNSQTGGLFGGATNSQTGGLFGGATNSQTGVLTKPN
EKNLCYAISNGTDFCIFELALTQRKLVKAGQLKPGAQGGLFGGATNSQTGGLFGGATNSQTGGLFGGATNSQTGGLF
GGATNSQTGGLFGGATNSQTGGLFGGATNSQTGGLFGGATNSQTGGLFGGATNSQTGGLFGGATNSQTGGLFGGATN
SQTGGLFGGATNSQTGGLFGGATNSQTGGLFGGATNSQTGGLFGGATNSQTGGLFGGATNSQTGGLFGGATNSQTGG
LFGGATNSQTGGLFGGATNSQTGGLFGGATNSQTGGLFGGATNSQTGGLFGGATNSQTGGLFGGATNSQTGGLFGGA
TNSQTSC

prf.GLFG$_{7x12}$
Plasmid: pSNG110

GSGGLFGGNTQPATGGLFGGNTQPATGGLFGGNTQPATGGLFGGNTQPATGGLFGGNTQPATGGLFGGNTQPATGGL
FGGNTQPATTSC

GLFG$_{52x12}$
Plasmid: pSNG036[#]

MQHHSHHGHHSHHGHHGHHGHHGHHGHHGSGLFGNTGGAPAGGLFGNTQTQQGGGLFGQPQQTQGGGLFGQTGATTG
GGLFGGATNTAPGGLFGGGGGNPTGGLFGGNNNQQTGGLFGQGTTQTGGGLFGAPQNNQGGGLFGGGTTTTGGGLFG
ANTQTGGGGLFGGPSQPTTAGLFGSNNPTTGGGLFGQPANTNNGGLFGGQTNNQASGLFGANNQPPTNGLFGNNNKP
QTAGLFGGATTTGNTGLFGGANNTGGGGLFGNNTNNPTGGLFGATNPAGGGGLFGGGATTGGGGLFGGGNTQTGGGL
FGTANTTTAGGLFGGGNTQPQNGLFGNNNTPATGGLFGQTNNAAPQGLFGGTNNNAASGLFGQKPASANGVLTKPNE
KNLCYAISNGTDFCIFELALTQRKLVKAGQLKPGAQAGGLFGQPAQNTQGGLFGGGGAATTPGLFGGAQNNTTGGLF
GGQNTQAGGGLFGAPNNAAATGLFGAGNANTQGGLFGAKPAATGGGLFGQPAQTQAGGLFGNTAQPAGGGLFGGATT
TPGGGLFGGNTAATGGGLFGGNTQGATGGLFGGQQPNNQGGLFGNTNANTGGGLFGGATTTTGGGLFGGSTGATGGG
LFGGASQPAAGGLFGGAAPQQNSGLFGGATAGQTGGLFGGATQQQGGGLFGQTASNPGGGLFGAANATTQPGLFGGN
NQAATSC

GAFG$_{52x12}$
Plasmid: pSNG072[#]

MQHHSHHGHHSHHGHHGHHGHHGHHGHHGSGAFGNTGGAPAGGAFGNTQTQQGGGAFGQPQQTQGGGAFGQTGATTG
GGAFGGATNTAPGGAFGGGGGNPTGGAFGGNNNQQTGGAFGQGTTQTGGGAFGAPQNNQGGGAFGGGTTTTGGGAFG
ANTQTGGGGAFGGPSQPTTAGAFGSNNPTTGGGAFGQPANTNNGGAFGGQTNNQASGAFGANNQPPTNGAFGNNNKP
QTAGAFGGATTTGNTGAFGGANNTGGGGAFGNNTNNPTGGAFGATNPAGGGGAFGGGATTGGGGAFGGGNTQTGGGA
FGTANTTTAGGAFGGGNTQPQNGAFGNNNTPATGGAFGQTNNAAPQGAFGGTNNNAASGAFGQKPASANGVLTKPNE
KNLCYAISNGTDFCIFELALTQRKLVKAGQLKPGAQAGGAFGQPAQNTQGGAFGGGGAATTPGAFGGAQNNTTGGAF
GGQNTQAGGGAFGAPNNAAATGAFGAGNANTQGGAFGAKPAATGGGAFGQPAQTQAGGAFGNTAQPAGGGAFGGATT
TPGGGAFGGNTAATGGGAFGGNTQGATGGAFGGQQPNNQGGAFGNTNANTGGGAFGGATTTTGGGAFGGSTGATGGG
AFGGASQPAAGGAFGGAAPQQNSGAFGGATAGQTGGAFGGATQQQGGGAFGQTASNPGGGAFGAANATTQPGAFGGN
NQAATTS

GXFG//L$_{52x12}$
Plasmid: pSNG087[#]

MQHHSHHGHHSHHGHHGHHGHHGHHGHHGSGGFGNTGLAPAGGTFGNTQLQQGGGQFGQPQLTQGGGAFGQTGLTTG
GGNFGGATLTAPGGGFGGGGLNPTGGNFGGNNLQQTGGTFGQGTLQTGGGNFGAPQLNQGGGTFGGGTLTTGGGQFG
ANTLTGGGGQFGGPSLPTTAGPFGSNNLTTGGGNFGQPALTNNGGNFGGQTLNQASGQFGANNLPPTNGKFGNNNLP
QTAGTFGGATLTGNTGNFGGANLTGGGGNFGNNTLNPTGGPFGATNLAGGGGTFGGGALTGGGGTFGGGNLQTGGGT
FGTANLTTAGGTFGGGNLQPQNGTFGNNNLPATGGNFGQTNLAAPQGNFGGTNLNAASGAFGQKPLSANGVLTKPNE
KNLCYAISNGTDFCIFELALTQRKLVKAGQLKPGAQAGGQFGQPALNTQGGAFGGGGLATTPGNFGGAQLNTTGGTF
GGQNLQAGGGNFGAPNLAAATGAFGAGNLNTQGGAFGAKPLATGGGQFGQPALTQAGGQFGNTALPAGGGTFGGATL
TPGGGAFGGNTLATGGGQFGGNTLGATGGPFGGQQLNNQGGAFGNTNLNTGGGTFGGATLTTGGGGFGGSTLATGGG
QFGGASLPAAGGPFGGAALQQNSGAFGGATLGQTGGQFGGATLQQGGGSFGQTALNPGGGAFGAANLTTQPGQFGGN
NLAATTS

GXFG//V$_{52x12}$
Plasmid: pSNG125
MQHHSHHGHHSHHGHHGHHGHHGHHGHHGSGGFGNTGVAPAGGTFGNTQVQQGGGQFGQPQVTQGGGAFGQTGVTTG
GGNFGGATVTAPGGGFGGGGVNPTGGNFGGNNVQQTGGTFGQGTVQTGGGNFGAPQVNQGGGTFGGGTVTTGGGQFG
ANTVTGGGGQFGGPSVPTTAGPFGSNNVTTGGGNFGQPAVTNNGGNFGGQTVNQASGQFGANNVPPTNGKFGNNNVP
QTAGTFGGATVTGNTGNFGGANVTGGGGNFGNNTVNPTGGPFGATNVAGGGGTFGGGAVTGGGGTFGGGNVQTGGGT
FGTANVTTAGGTFGGGNVQPQNGTFGNNNVPATGGNFGQTNVAAPQGNFGGTNVNAASGAFGQKPVSANGVLTKPNE
KNLCYAISNGTDFCIFELALTQRKLVKAGQLKPGAQAGGQFGQPAVNTQGGAFGGGGVATTPGNFGGAQVNTTGGTF
GGQNVQAGGGNFGAPNVAAATGAFGAGNVNTQGGAFGAKPVATGGGQFGQPAVTQAGGQFGNTAVPAGGGTFGGATV
TPGGGAFGGNTVATGGGQFGGNTVGATGGPFGGQQVNNQGGAFGNTNVNTGGGTFGGATVTTGGGGFGGSTVATGGG
QFGGASVPAAGGPFGGAAVQQNSGAFGGATVGQTGGQFGGATVQQGGGSFGQTAVNPGGGAFGAANVTTQPGQFGGN
NVAATSC

GLAG$_{52x12}$
Plasmid: pSNG123
GSGLAGNTGGAPAGGLAGNTQTQQGGGLAGQPQQTQGGGLAGQTGATTGGGLAGGATNTAPGGLAGGGGNPTGGLA
GGNNNQQTGGLAGQGTTQTGGGLAGAPQNNQGGGLAGGGTTTTGGGLAGANTQTGGGGLAGGPSQPTTAGLAGSNNP
TTGGGLAGQPANTNNGGLAGGQTNNQASGLAGANNQPPTNGLAGNNNKPQTAGLAGGATTTGNTGLAGGANNTGGGG
LAGNNTNNPTGGLAGATNPAGGGGLAGGGATTGGGGLAGGGNTQTGGGLAGTANTTTAGGLAGGGNTQPQNGLAGNN
NTPATGGLAGQTNNAAPQGLAGGTNNNAASGLAGQKPASANGVLTKPNEKNLCYAISNGTDFCIFELALTQRKLVKA
GQLKPGAQAGGLAGQPAQNTQGGLAGGGGAATTPGLAGGAQNNTTGGLAGGQNTQAGGGLAGAPNNAAATGLAGAGN
ANTQGGLAGAKPAATGGGLAGQPAQTQAGGLAGNTAQPAGGGLAGGATTTPGGGLAGGNTAATGGGLAGGNTQGATG
GLAGGQQPNNQGGLAGNTNANTGGGLAGGATTTTGGGLAGGSTGATGGGLAGGASQPAAGGLAGGAAPQQNSGLAGG
ATAGQTGGLAGGATQQQGGGLAGQTASNPGGGLAGAANATTQPGLAGGNNQAATSC

GLLG$_{52x12}$
Plasmid: pSNG124
GSGLLGNTGGAPAGGLLGNTQTQQGGGLLGQPQQTQGGGLLGQTGATTGGGLLGGATNTAPGGLLGGGGNPTGGLL
GGNNNQQTGGLLGQGTTQTGGGLLGAPQNNQGGGLLGGGTTTTGGGLLGANTQTGGGGLLGGPSQPTTAGLLGSNNP
TTGGGLLGQPANTNNGGLLGGQTNNQASGLLGANNQPPTNGLLGNNNKPQTAGLLGGATTTGNTGLLGGANNTGGGG
LLGNNTNNPTGGLLGATNPAGGGGLLGGGATTGGGGLLGGGNTQTGGGLLGTANTTTAGGLLGGGNTQPQNGLLGNN
NTPATGGLLGQTNNAAPQGLLGGTNNNAASGLLGQKPASANGVLTKPNEKNLCYAISNGTDFCIFELALTQRKLVKA
GQLKPGAQAGGLLGQPAQNTQGGLLGGGGAATTPGLLGGAQNNTTGGLLGGQNTQAGGGLLGAPNNAAATGLLGAGN
ANTQGGLLGAKPAATGGGLLGQPAQTQAGGLLGNTAQPAGGGLLGGATTTPGGGLLGGNTAATGGGLLGGNTQGATG
GLLGGQQPNNQGGLLGNTNANTGGGLLGGATTTTGGGLLGGSTGATGGGLLGGASQPAAGGLLGGAAPQQNSGLLGG
ATAGQTGGLLGGATQQQGGGLLGQTASNPGGGLLGAANATTQPGLLGGNNQAATSC

GLLG//L$_{52x12}$
Plasmid: pSNG131[#]
MQHHSHHGHHSHHGHHGHHGHHGHHGHHGSGLLGNTGLAPAGGLLGNTQLQQGGGLLGQPQLTQGGGLLGQTGLTTG
GGLLGGATLTAPGGLLGGGGLNPTGGLLGGNNLQQTGGLLGQGTLQTGGGLLGAPQLNQGGGLLGGGTLTTGGGLLG
ANTLTGGGGLLGGPSLPTTAGLLGSNNLTTGGGLLGQPALTNNGGLLGGQTLNQASGLLGANNLPPTNGLLGNNNLP
QTAGLLGGATLTGNTGLLGGANLTGGGGLLGNNTLNPTGGLLGATNLAGGGGLLGGGALTGGGGLLGGGNLQTGGGL
LGTANLTTAGGLLGGGNLQPQNGLLGNNNLPATGGLLGQTNLAAPQGLLGGTNLNAASGLLGQKPLSANGVLTKPNE
KNLCYAISNGTDFCIFELALTQRKLVKAGQLKPGAQAGGLLGQPALNTQGGLLGGGGLATTPGLLGGAQLNTTGGLL
GGQNLQAGGGLLGAPNLAAATGLLGAGNLNTQGGLLGAKPLATGGGLLGQPALTQAGGLLGNTALPAGGGLLGGATL
TPGGGLLGGNTLATGGGLLGGNTLGATGGLLGGQQLNNQGGLLGNTNLNTGGGLLGGATLTTGGGLLGGSTLATGGG
LLGGASLPAAGGLLGGAALQQNSGLLGGATLGQTGGLLGGATLQQGGGLLGQTALNPGGGLLGAANLTTQPGLLGGN
NLAATSC

His-tag-free Mac98A ΔGLEBS
Plasmid: pSNG122

GSMFGNTGGGGLFGNTQTQQTGGGLFGQPQQTQFGQTGATGGGLFGGATNTFGGGGGGGLFGGNNNQQTNPTAGGGI
FGQGTTGLGGAPAQTGGGLFGAPQNNQGGGLFGGGTTTGGGMFGNQANTQTGGGGLFGGPSQPTTQPPAFSLNNPTT
GGGGLFGQPANTMGGNNGGLFGGQTNSFGANNNMLGNNNRPQGAGIFGGATTTAPTGNTGMFGGIGANNGGGGLFGM
NNTNTNPTGGFGATNPTAGGGGLFGGGATTTGGGGLFGGGNTQGGGLLGTANTTAGGLLGGGFNMNNNTGGILGQTN
NQFGLGSFGTNNNAAAAPFQPKASANGVLVKAGQLKPGAQQAGGMFGQPAQGGNGLFGGGGAATTTPFGGAQNGNLF
GGQNTQAQGGGLFGAPVNNAATGAGGGLFGAKPAATTTGGGLFGQMPAQTGGFLGNTATQPAGGGLFGGATTTQAPG
GGGGGGLFGGNTTAATTGGGLFGGNTQTGGATGGLFGGQQPNNQGGLFLNTGNANNANTGGGLFGGATTTPATGGGL
FGGSTNTQPGLATGGGLFGNNQGASQPAAQGGLFGGAAPQQNSLFGGATAGGQTGGLFGGATGATQQQGGGLFGQTA
SNPTQGGGLFGAANPGLGGAAATSC

Protein name	Plasmid	Encoding for	Used in figures	Reference
His_{18}-*Tt*MacNup98A (Mac98A) FG domain	pHBS418	His_{18}-*Tt*MacNup98A$_{1-666}$-Cys	1, 2, 3, S2	Schmidt and Görlich, 2015[4]
prf.GLFG$_{52x12}$*	pSNG064	His_{14}-ZZ-*sc*SUMO-prf.GLFG$_{52x12}$	1-7, S1, S3, S4, S5	this study
prf.GLFG$_{52x12}$-Cys*	pSNG102	His_{14}-ZZ-*sc*SUMO-prf.GLFG$_{52x12}$-Cys	2a, 5b, S1	this study
His_{18}-Pro-free prf.GLFG$_{52x12}$	pSNG037	His_{18}- Pro-free_ prf.GLFG$_{52x12}$-Cys	2b	Ng et al., 2021[5]
prf.GLFG$_{7x12}$*	pSNG110	His_{14}-ZZ-*sc*SUMO-prf.GLFG$_{7x12}$	4	this study
His_{18}-GLFG$_{52x12}$	pSNG036	His_{18}-GLFG$_{52x12}$	7a, S7	Ng et al., 2021[5]
GLFG$_{52x12}$-Cys*	pSNG114	His_{14}-ZZ-*sc*SUMO-GLFG$_{52x12}$-Cys	7b, c	this study
His_{18}-GAFG$_{52x12}$	pSNG072	His_{18}- GAFG$_{52x12}$	7a, S6	this study
GAFG$_{52x12}$-Cys*	pSNG112	His_{14}-ZZ-*sc*SUMO-GAFG$_{52x12}$-Cys	7b, c	this study
His_{18}-GXFG//L$_{52x12}$	pSNG087	His_{18}-GXFG//L$_{52x12}$	7a, S7	this study
GXFG//L$_{52x12}$-Cys*	pSNG113	His_{14}-ZZ-*sc*SUMO-GxFG//L$_{52x12}$-Cys	7b, c	this study
His_{18}-GXFG//V$_{52x12}$	pSNG125	His_{18}-GxFG//V$_{52x12}$	7a	this study
GLAG$_{52x12}$*	pSNG123	His_{14}-ZZ-*sc*SUMO-GLAG$_{52x12}$-Cys	7a, S6	this study
GLLG$_{52x12}$*	pSNG124	His_{14}-ZZ-*sc*SUMO-GLLG$_{52x12}$-Cys	7a, S6	this study
His_{18}-GLLG//L$_{52x12}$	pSNG131	His_{18}-GLLG//L$_{52x12}$	7a, S7	this study
GLLG//L$_{52x12}$-Cys*	pSNG132	His_{14}-ZZ-*sc*SUMO-GLLG//L$_{52x12}$-Cys	7b, c	this study
Mac98A ΔGLEBS*	pSNG122	His_{14}-ZZ-*sc*SUMO-*Tt*MacNup98A$_{1-666\ \Delta 336-365}$-Cys	S2	this study
rat (*Rattus norvegicus*) NTF2	pDG2121	*r*NTF2	1a	Frey et al., 2018[6]
mCherry*	pSF779	His_{14}-TEV-mCherry-Cys	1a, S7	Schmidt & Görlich, 2015[4]

Supplementary Table 1: Proteins and corresponding bacterial expression constructs used in this study. Plasmid numbers are unique identifiers. * indicates that a histidine-tag-cleaved (by SUMO/ TEV) version of the protein was used.

Components	Final conc.
Na_2HPO_4	100 mM
KH_2PO_4	20 mM
$MgSO_4$	2 mM
$CaCl_2$	0.2 mM
$NTA \cdot Na_2$	500 μM
$FeCl_3$	100 μM
$ZnCl_2$	10 μM
$MnCl_2$	10 μM
H_3BO_4	5 μM
$NiSO_4$	0.5 μM
$(NH_4)_6Mo_7O_{24}$	0.5 μM
Na_2SeO_3	0.5 μM
Na_2WO_4	0.5 μM
$CoSO_4$	1 μM
$CuSO_4$	1 μM
Biotin	1 mg/L
Thiamine	1 mg/L
$^{15}NH_4Cl$ (Sigma-Aldrich, Germany)	1.1 g/L
D-Glucose U-$^{13}C_6$ (Cambridge Isotope Laboratories, USA)	6.0 g/L

Supplementary Table 2: Composition of M9 medium supplemented with trace elements and isotope labels for bacterial cultures.

References

1. Shaka AJ, Keeler J, Frenkiel T, Freeman R. An Improved Sequence for Broad-Band Decoupling - Waltz-16. *J Magn Reson* **52**, 335-338 (1983).

2. Shaka AJ, Keeler J, Freeman R. Evaluation of a New Broad-Band Decoupling Sequence - Waltz-16. *J Magn Reson* **53**, 313-340 (1983).

3. Williamson MP. Using chemical shift perturbation to characterise ligand binding. *Progress in nuclear magnetic resonance spectroscopy* **73**, 1-16 (2013).

4. Schmidt HB, Görlich D. Nup98 FG domains from diverse species spontaneously phase-separate into particles with nuclear pore-like permselectivity. *eLife* **4**, (2015).

5. Ng SC, Güttler T, Görlich D. Recapitulation of selective nuclear import and export with a perfectly repeated 12mer GLFG peptide. *Nat Commun* **12**, 4047 (2021).

6. Frey S, *et al.* Surface Properties Determining Passage Rates of Proteins through Nuclear Pores. *Cell* **174**, 202-217 e209 (2018).
