# Supplementary material for: Atomic resolution dynamics of cohesive interactions in phase-separated Nup98 FG domains
Source: Nat Commun. 2022 Mar 21;13:1494. doi: 10.1038/s41467-022-28821-8 (PMC8938434; doi:10.1038/s41467-022-28821-8)
Supplement: Supplementary file 3 — Description of additional Supplementary File [file 41467_2022_28821_MOESM3_ESM.pdf]

<u>Description of additional supplementary data files</u>

Supplementary Software : The Jupyter notebook (software) for fitting TRACT data.
