# Supplementary material for: Atomic resolution dynamics of cohesive interactions in phase-separated Nup98 FG domains
Source: Nat Commun. 2022 Mar 21;13:1494. doi: 10.1038/s41467-022-28821-8 (PMC8938434; doi:10.1038/s41467-022-28821-8)
Supplement: Supplementary file 6 — Reporting Summary [file 41467_2022_28821_MOESM6_ESM.pdf]

# nature portfolio

Corresponding author(s): Dirk Görlich, Loren B. Andreas

Last updated by author(s): Dec 30, 2021

# Reporting Summary

Nature Portfolio wishes to improve the reproducibility of the work that we publish. This form provides structure for consistency and transparency in reporting. For further information on Nature Portfolio policies, see our Editorial Policies and the Editorial Policy Checklist.

## Statistics

For all statistical analyses, confirm that the following items are present in the figure legend, table legend, main text, or Methods section.

| n/a | Confirmed | |
|---|---|---|
| ☐ | ☒ | The exact sample size (*n*) for each experimental group/condition, given as a discrete number and unit of measurement |
| ☐ | ☒ | A statement on whether measurements were taken from distinct samples or whether the same sample was measured repeatedly |
| ☒ | ☐ | The statistical test(s) used AND whether they are one- or two-sided<br>*Only common tests should be described solely by name; describe more complex techniques in the Methods section.* |
| ☒ | ☐ | A description of all covariates tested |
| ☒ | ☐ | A description of any assumptions or corrections, such as tests of normality and adjustment for multiple comparisons |
| ☐ | ☒ | A full description of the statistical parameters including central tendency (e.g. means) or other basic estimates (e.g. regression coefficient) AND variation (e.g. standard deviation) or associated estimates of uncertainty (e.g. confidence intervals) |
| ☒ | ☐ | For null hypothesis testing, the test statistic (e.g. *F*, *t*, *r*) with confidence intervals, effect sizes, degrees of freedom and *P* value noted<br>*Give P values as exact values whenever suitable.* |
| ☒ | ☐ | For Bayesian analysis, information on the choice of priors and Markov chain Monte Carlo settings |
| ☒ | ☐ | For hierarchical and complex designs, identification of the appropriate level for tests and full reporting of outcomes |
| ☒ | ☐ | Estimates of effect sizes (e.g. Cohen's *d*, Pearson's *r*), indicating how they were calculated |

*Our web collection on statistics for biologists contains articles on many of the points above.*

## Software and code

Policy information about availability of computer code

| | |
|---|---|
| Data collection | Leica Application Suite X 3.3.0, Wyatt Technologies Dynamics 7.1.5, Topspin 4.0.8. All software used is commercially available. |
| Data analysis | Microsoft Excel version 16.42, Jupyter Notebook. All software used is commercially available. The Jupyter notebook for fitting TRACT data is deposited as a supplementary file. |

For manuscripts utilizing custom algorithms or software that are central to the research but not yet described in published literature, software must be made available to editors and reviewers. We strongly encourage code deposition in a community repository (e.g. GitHub). See the Nature Portfolio guidelines for submitting code & software for further information.

## Data

Policy information about availability of data

All manuscripts must include a data availability statement. This statement should provide the following information, where applicable:

- Accession codes, unique identifiers, or web links for publicly available datasets
- A description of any restrictions on data availability
- For clinical datasets or third party data, please ensure that the statement adheres to our policy

A source data file is provided with this article. The data that support the findings of this study are provided in the Supplementary Information or Source Data file or are available from the corresponding authors upon reasonable request.

# Field-specific reporting

Please select the one below that is the best fit for your research. If you are not sure, read the appropriate sections before making your selection.

- [x] Life sciences
- [ ] Behavioural & social sciences
- [ ] Ecological, evolutionary & environmental sciences

For a reference copy of the document with all sections, see nature.com/documents/nr-reporting-summary-flat.pdf

# Life sciences study design

All studies must disclose on these points even when the disclosure is negative.

| | |
|---|---|
| Sample size | Sample sizes were not predetermined by statistical methods, but determined by the variability of the replicates. E.g., in FG particle FRAP assays, S.D. were typically <15% between replicates as reported in this study and previously (Ng SC, Güttler T, Görlich D. 2021 Recapitulation of selective nuclear import and export with a perfectly repeated 12mer GLFG peptide. Nat Commun 12, 4047), and thus the lower limit of the sample size was set to 5. Note that the overall trend is clear and reproducible for both FRAP and DLS measurements from 5 or 3 replicates, respectively. |
| Data exclusions | No data were excluded. |
| Replication | Numbers of replicates for FRAP and DLS experiments were as stated in the figures. Analyses of phase separation were repeated three times for each. The other experiments were repeated as reported in the main text (Statistics and Reproducibility/ Method sections). Attempts at replication were successful. |
| Randomization | This is not applicable to our study, because our samples were not taken from a preexisting distribution but were designed and produced for specific purposes and specific measurements. |
| Blinding | Investigators were not blinded during the analyses, because otherwise the investigators would have been unable to apply the relevant measurement settings to specific samples. Moreover, there are fundamental differences in the readouts of our samples (e.g., wild-type FG domains versus perfectly repetitive FG repeats) and thus the influence of subjectivity is not significant enough to require blinding. |

# Reporting for specific materials, systems and methods

We require information from authors about some types of materials, experimental systems and methods used in many studies. Here, indicate whether each material, system or method listed is relevant to your study. If you are not sure if a list item applies to your research, read the appropriate section before selecting a response.

## Materials & experimental systems

| n/a | Involved in the study | |
|---|---|---|
| x | | Antibodies |
| x | | Eukaryotic cell lines |
| x | | Palaeontology and archaeology |
| x | | Animals and other organisms |
| x | | Human research participants |
| x | | Clinical data |
| x | | Dual use research of concern |

## Methods

| n/a | Involved in the study | |
|---|---|---|
| x | | ChIP-seq |
| x | | Flow cytometry |
| x | | MRI-based neuroimaging |
